# Transcranial Pulse Stimulation (TPS) suitable add-on treatment of patients with psychiatric disorders?

**DOI:** 10.1192/j.eurpsy.2025.1634

**Published:** 2025-08-26

**Authors:** V. Rößner-Ruff, C. A. Penkov, J. Krieger, K. Friedrich, M. Ziegenbein

**Affiliations:** 1 Psychiatry and Psychotherapy, Wahrendorff Clinic, Sehnde, Germany

## Abstract

**Introduction:**

Neurostimulation procedures have also been in use for some time to treat neurological and neuropsychiatric disorders. In the field of non-invasive brain stimulation, a number of methods have been the subject of investigation, including transcranial magnetic stimulation (TMS), transcranial electrical stimulation (tES), and transcranial pulse stimulation (TPS). Although TMS and tES have been the subject of more extensive investigation, particularly in the context of Alzheimer’s disease (AD) and depression, there remains a pressing need for research into TPS. Some studies have demonstrated favorable outcomes when these methods were used. Nevertheless, these methodes have yet to be incorporated into clinical practice, largely due to the inability to conclusively assess and confirm their effectiveness, which is influenced by a number of factors.

**Objectives:**

TPS as a method of neurostimulation will be presented. Results of a recent study using TPS in patients with mild to moderate Alzheimer’s disease will also be presented. The aim of the study is to investigate whether cognitive performance is significantly increased and depressive symptom severity is significantly reduced. Furthermore, a research proposal for the use of TPS in patients diagnosed with a depressive disorder will be presented.

**Methods:**

The cognitive performance (MoCA) and depressive symptoms (GDS) of patients with early- or late-onset, mild or moderate AD will be assessed at baseline (t1), three months (t2) and six months (t3) following treatment with TPS. In the context of this longitudinal study, which is designed with a single center, the participants will be recruited in an outpatient setting within a specialized psychiatric-psychotherapeutic clinic in Lower Saxony, Germany.

**Results:**

Repeated measures ANOVA; Mean scores MoCA t1 = 13,9 vs. t2 = 14,1 vs. t3 = 14,5 (F(2, 66) = .749, *n.s.*). Mean scores GDS t1 = 9,5 vs. t2 = 7,6 vs. t3 = 6,8 (F(2, 32 = 4.410, *p ˂ .05*). n = 34. Preliminary data for the study of TPS in AD, study is being continued. Research proposal for the use of TPS in patients diagnosed with a depressive disorder will also be presented.

**Conclusions:**

As in other studies, remarkable effects were shown with the use of TPS in patients with a mild to moderate AD. The results may indicate that the cognitive performance can be stabilized and the depressive symptom severity can be reduced. It is important to note that the results are based on a relatively small sample size and lack control conditions, which may limit the generalizability of the findings. Further research is required to evaluate the effectiveness of treatment of TPS in AD and to assess the potential suitability of neurostimulation as an add-on therapy, complementing previous drug and non-drug approaches. Further research is also required in the use of TPS in depression given the dearth of scientifically reliable findings on this topic.

**Disclosure of Interest:**

None Declared

